# Video Endoscopy for Laser Photoresection in Tracheobronchial Pathology: Some Considerations After 9 Years Experience With 2105 Treatments

**DOI:** 10.1155/DTE.2.79

**Published:** 1995

**Authors:** Jose Pablo Diaz-Jimenez, Jose Ignacio Martinez Ballarin, Eva Farrero Muñoz, Kevin L. Kovitz, Maria Jesus Castro Serrano, K. Shirakura

**Affiliations:** 1 Endoscopy Laser Unit, Pneumology Service Hospital Duran y Reynals Ciudad Sanitaria y Universitaria de Bellvitge Hospitalet de Llobregat Barcelona 08907 Spain; 2 Section of Pulmonmary and Critical Care Medicine Tulane University Medical Center New Orleans Louisiana USA; 3 Department of Orthopaedic Surgery Gunma University School of Medicine 3-39-22 Showa-machi Gunma-ken Maebashi-shi 371 Japan

## Abstract

Between 1984 and 1993 we performed 2105 laser treatments in 1210 patients: 52% of treatments were done for malignant pathology, 45% for benign tracheal stenoses and 3% were in a miscellaneous group. The procedure was carried out with a rigid bronchoscope under general anaesthesia. In patients with malignant tumors, it is a good palliative treatment—safe, well tolerated and with immediate results; it can be repeated as many times as needed with and is well accepted by the patient. In patients without tumors, this method avoids emergency tracheotomies. The long term results are now under evaluation.


*Diagnostic and Therapeutic Endoscopy*, 1995, Vol. 2, pp. 79–87
Reprints available directly from the publisher
Photocopying permitted by license only

© 1995 Harwood Academic Publishers GmbH
Printed in Singapore

### Video Endoscopy for Laser Photoresection in Tracheobronchial Pathology: Some Considerations After 9 Years Experience with 2105 Treatments

JOSE PABLO DIAZ-JIMENEZ, JOSE IGNACIO MARTINEZ BALLARIN, EVA FARRERO MUÑOZ, KEVIN L. KOVITZ** and MARIA JESUS CASTRO SERRANO

*Endoscopy Laser Unit, Pneumology Service, Hospital Duran y Reynals, Ciudad Sanitaria y Universitaria de Bellvitge, Hospitalet de Llobregat 08907, Barcelona Spain, and Section of Pulmonmary and Critical Care Medicine, Tulane University Medical Center, New Orleans, Louisiana*

*(Received December 30, 1994; in final form April 26, 1995)*

Between 1984 and 1993 we performed 2105 laser treatments in 1210 patients: 52% of treatments were done for malignant pathology, 45% for benign tracheal stenoses and 3% were in a miscellaneous group. The procedure was carried out with a rigid bronchoscope under general anaesthesia. In patients with malignant tumors, it is a good palliative treatment—safe, well tolerated and with immediate results; it can be repeated as many times as needed with and is well accepted by the patient. In patients without tumors, this method avoids emergency tracheotomies. The long term results are now under evaluation.

**KEY WORDS:** Nd-YAG Laser, photoresection, tracheobronchial stent

### INTRODUCTION

Since the 1930's, there has been a clear desire to eliminate, through endoscopic methods, large tumoral or iatrogenic obstructions of the airways. In 1935, Kramer and Som (1) published the first results of endoscopic resection in 20 patients with bronchial adenoma, observing years later that of the 14 patients that they had considered adequately treated, 50% were cured or free of symptoms (2). Further attempts to endoscopically resect malignant lesions were soon abandoned because postulated improvements in ventilation were offset by the significant risk of hemorrhagic complications (3). More recently, transbronchoscopic cryosurgery (4) and intraluminal radiotherapy (5) have been tried with limited benefits in the removal of the bronchial obstruction.

Endobronchial laser treatment has shown promise since the first report of Strong and colleagues in 1974 (6). Since then the use of lasers in the tracheobronchial tree has continued to advance. Since the 1980's, laser bronchoscopy has reached its apogee, as demonstrated in the extensive published series (7–10). This report presents some considerations after 9 years experience with 2015 procedures.

### MATERIALS AND METHODS

Between 1984 and 1993 we performed a total of 2105 treatments in 1210 patients (Fig. 1). These consisted of 1094 (52%) treatments for tumors, 947 (45%) for tracheal stenosis and 64 (3%) treatments for a miscellaneous group that included granulomas, small recurrences of bronchial carcinoma on suture scars, management of local bleeding and dislodgement of impacted foreign bodies. Most of the procedures were carried out with a rigid bronchoscope and under general anesthesia.

### Instruments

#### *Transmission Apparatus of the Laser*

The Nd-YAG laser is well suited to the treatment of tracheobronchial pathology because of its tissue penetration and its great capacity for coagulation. Its energy source

Address for correspondence: K. Shirakura M.D., Ph.D., Department of Orthopaedic Surgery, Gunma University School of Medicine, 3-39-22 Showa-machi, Maebashi-shi, Gunma-ken, Japan 371.

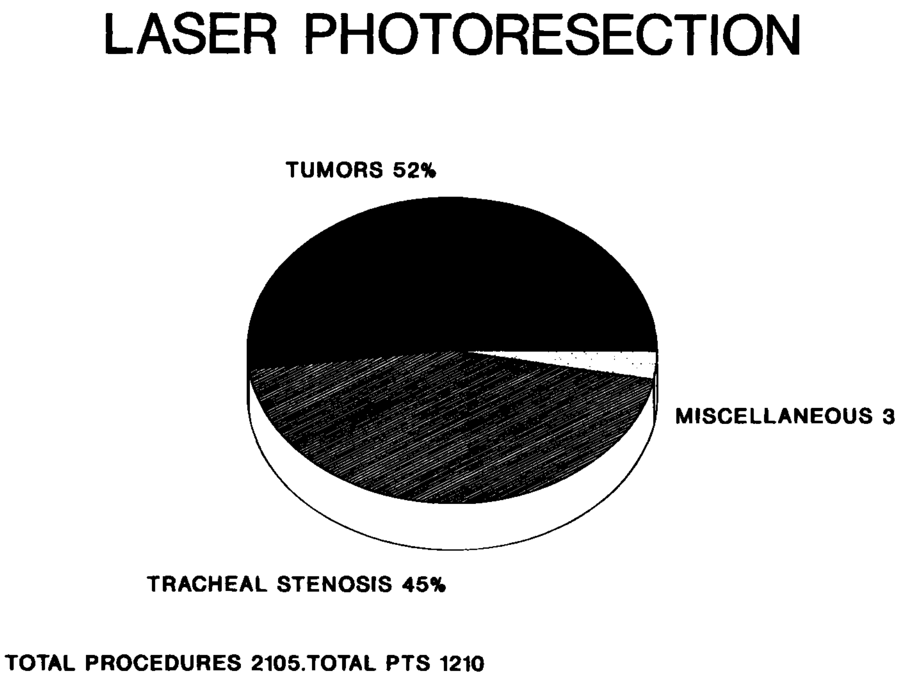

**Figure 1** Total Experience.

can emit energy in the range of 0 to 100 watts. Emission of the laser beam is controlled by a foot pedal operated by the bronchoscopist.

In our system, the laser is transmitted from the generator to the tissues through a fiber 0.6 mm in diameter, surrounded by a Teflon sleeve through which gas (nitrogen) is passed to obtain cooling and to prevent combustion. Since the light of the Nd-YAG laser is invisible to the human eye, a coaxial red helium-neon laser is used for targeting. The distal end is protected by a metallic tube of stainless steel to avoid ignition by overheating during treatment. Recently, disposable fibers have been developed which allow either contact or non-contact applications. These fibers are able to cut (approximating the $CO_2$ effect in the tissue) and to coagulate.

### Endoscopes

The rigid bronchoscope (Fig. 2) we employ for photoresection has a principal tube of 14 mm in diameter. Through this tube the following can be introduced: laser transmission fiber, forceps and narrower diameter bronchial tubes. This gives the bronchoscopist access to 4 to 10 mm diameter airways. At the main opening, a swiveling head is connected with a lateral port for ventilation. Two other lateral apertures allow the introduction of semirigid aspiration catheters and the laser fiber. An optional guide to fix the laser fiber can be used to improve the precision of the beam. A fiberoptic bronchoscope with an aspiration channel greater than 2.1 mm in diameter can be used in small resections, but is more commonly introduced through the rigid bronchoscope to clear the bronchial tree of blood, secretions or detritus at the end of the intervention.

### Method

The majority of the treatments are done with a rigid bronchoscope and under general anesthesia. Adequate anesthesia is fundamental for the success of these treatments. Since we ventilate the patient through the rigid bronchoscope with an open circuit, the patient is ideally asleep, breathing spontaneously, with the cough reflex abolished. It is extremely important to avoid oxygen concentrations over 50% because of the danger of explosion or ignition during the bursts of laser emissions.

Once the patient is anesthetized and appropriate monitoring is established, we proceed to intubation and ventilation through the rigid bronchoscope. We then inspect the airway. Care is taken to avoid contact with the lesion at this point so we can best define the limits of the lesion and avoid hemorrhage, which would obscure the field. A thorough understanding of thoracic anatomy is necessary to prevent iatrogenic damage to important surrounding structures. The limits of the lesion are defined by imaging studies (i.e., X-rays, tomograms, computed tomogra-

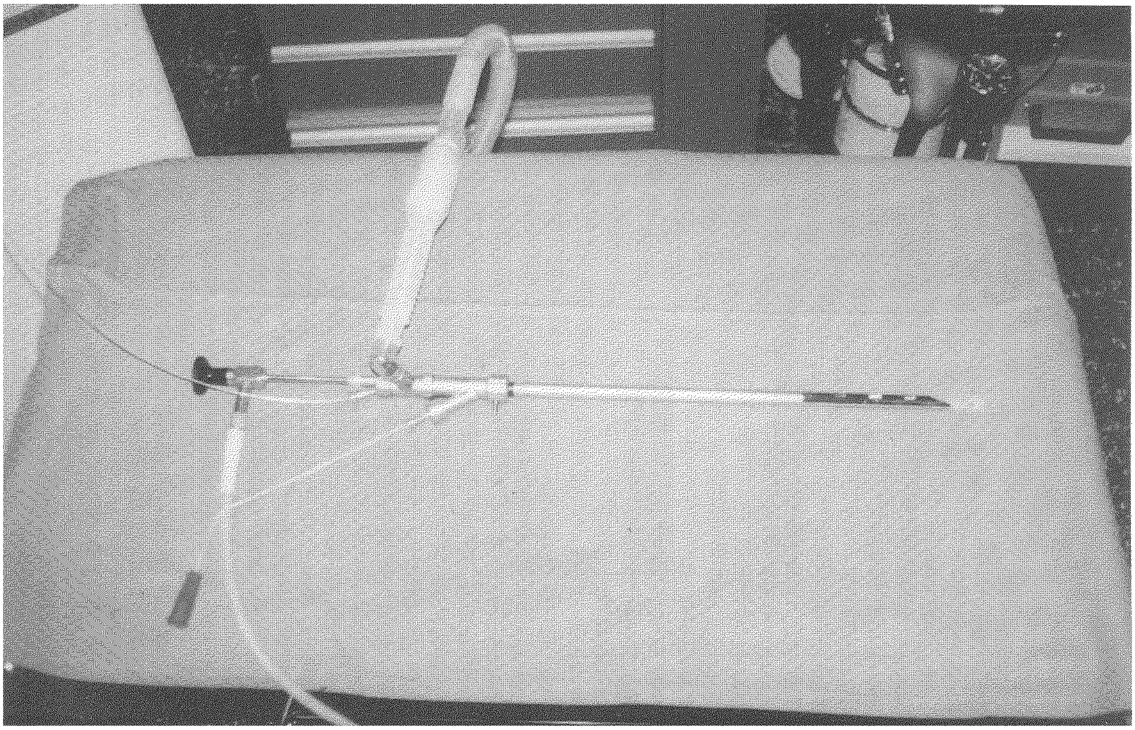

**Figure 2** Rigid bronchoscope for laser treatments.

phy scans, esophograms, etc.) performed in addition to routine preoperative studies.

The first few bursts of laser photoradiation are used to estimate the tissue energy absorption characteristics and the depth of the laser penetration. We vary the power and the duration of the laser shots as the treatment progresses. We use short bursts at high energy to resect tumor and longer bursts at low energy to coagulate. We never use a continuous pattern of firing as we consider this to be unsafe. The beam of the Nd-YAG laser, which is always coaxial with the fiber could damage surrounding structures. The tip of the bronchoscope can be used as an additional aid to ablate and remove tissue incised by the laser beam.

### RESULTS

Our total experience include 2105 resections in 1210 patients. As laser photoresection is almost always a palliative rather than a curative procedure, immediate results were classified as excellent, good or poor according to initial improvement of symptoms and airway diameter. Excellent results were achieved in 71.6% of tracheal tumors. Of the patients with tracheal stenosis, 35% had recurrences and underwent a repeat laser photoresection, surgery or prosthesis placement (Fig. 3). In the past 2 years, we placed a total of 125 stents for both malignant tumors (84/68%) and benign tracheal stenosis (41/32%) (Fig. 4). The most significant complication of stenting was migration which was greater with underlying benign pathology than with underlying malignant pathology.

### DISCUSSION

### Malignant Tumor Pathology

Laser photoresection of tumors is limited to inoperable cases. These include cases of tumor that has gone beyond the limits of resection or in which the condition of the patient precludes surgery. The objective always has to be relief of obstruction (Fig. 5), regardless of the histological type of tumor. The reestablishment of the airways by photoresection represents the most important palliative treatment because it can immediately correct ventilatory compromise. It also facilitates drainage of the secretions that can cause infection, and it can control hemoptysis (11–14). The therapy helps retard the endobronchial progression of the neoplasia within the airways and especially towards the trachea or contralateral side. In patients with malignant tumors with peripheral obstruction and consequent pneumonitis or abscess, the widening of the

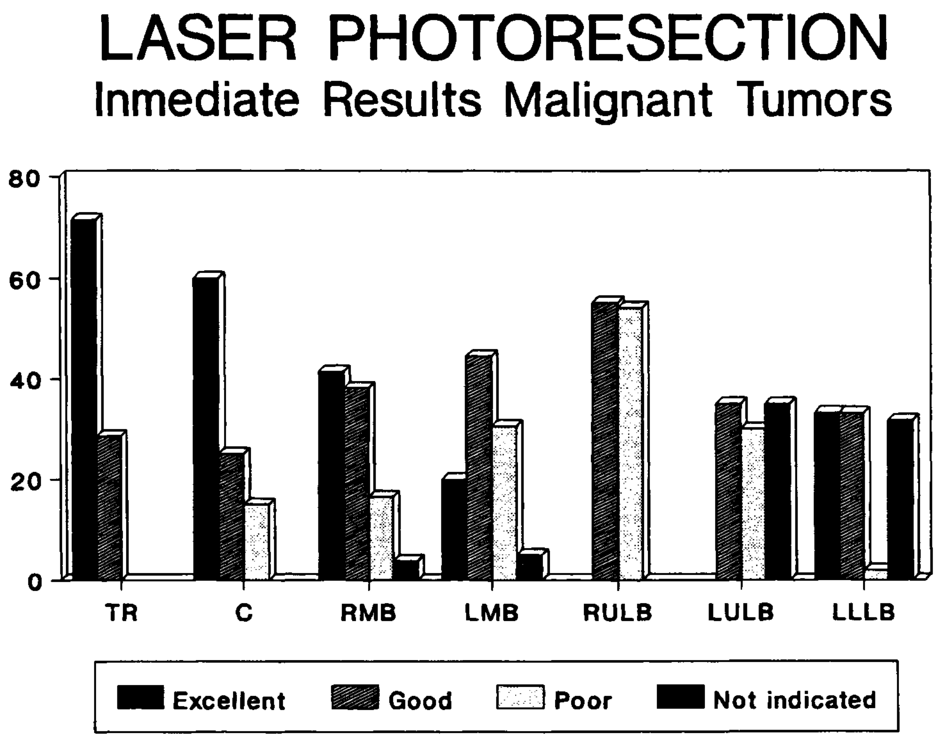

**3A** PATIENTS 720 PROCEDURES 1094

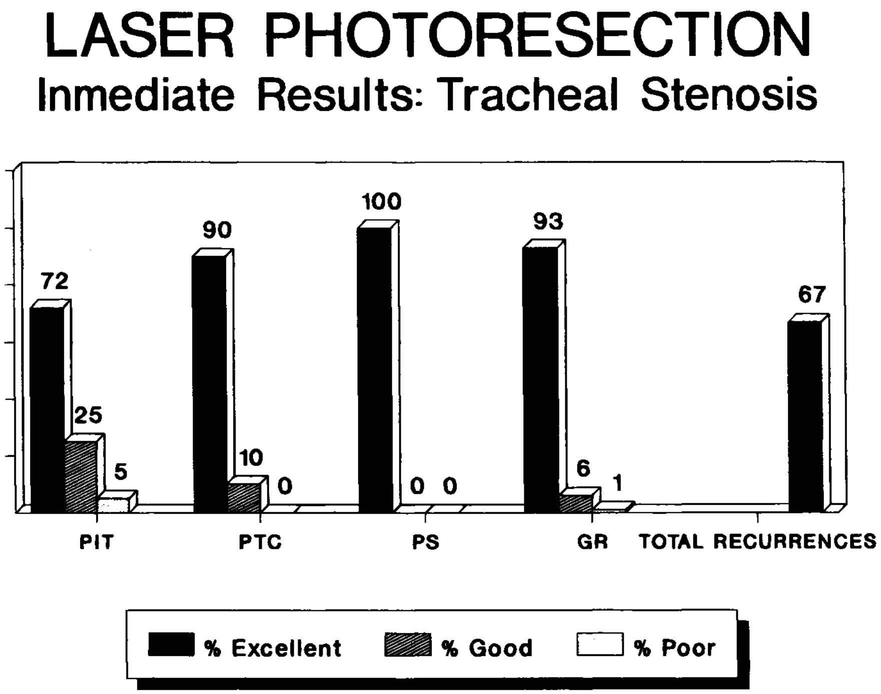

**3B** 440 patients. 947 procedures.

**Figure 3** Immediate results for malignant tumors.

PATIENTS

90 PATIENTS/125 PROSTHESES

| MALIGNANT | BENIGN |
|---|---|
| 60 P.(54 M, 6 W) | 30 P.(19 M, 11 W) |
| Mean Age 62, (r 35-91) | Mean Age 51, (r 18-78) |
| 84 PROSTHESES | 41 PROSTHESES |

**Figure 4** Immediate results for tracheal stenosis.

bronchus with laser therapy with resulting improved drainage can relieve infection and is beneficial even though we know there is no improvement in the ventilation/perfusion index (15). In cases of large central tumors that invade the pulmonary artery, the improvement of ventilation without perfusion results in a poor functional result. Nevertheless, the treatment is indicated to retard the growth of the tumor toward the trachea or toward the contralateral side in order to avoid death by asphyxia.

### Low Grade Malignant Tumors

In cases of carcinoid tumors and tumors deriving from bronchial glands (i.e. adenoid cystic carcinoma and mucoepidermoid tumor), laser photeresection is reserved for inoperable tumors. The most serious risk in the endobronchial resection of carcinoid tumor is hemorrhage. This can be reduced by using the laser for coagulation and other local approaches, such as compression with the rigid bronchoscope. With cylindroma, laser resection may be the only means of relief of obstruction since these tumors are found with greater frequency in the trachea, and at times may even extensively involve both main bronchi. Their tendency to be locally invasive is related to frequent recurrence. A mucoepidermoid tumor, because of its polypoid form, can be confused macroscopically with a benign tumor. Its resection is not associated with many complications (16).

### Benign Tumors

Benign tumors constitute the best indication for photoresection. Unfortunately, the frequency of benign tumors is less than malignant tumors. They include hamartomas, lipomas, chondromas, leiomyomas, etc., and represent a rare group in terms of endobronchial pathology. Their external appearance is similar: pale, small, round, poorly vascularized and, in general, pedunculated. Resection is simple and almost always definitive. The recurrence rate is low (8,9).

Diffuse tracheobronchial amyloidosis is a unique condition. Photoresection is difficult because of the diffuse extension and the tendency for bleeding. Repeat procedures are often needed. Diffuse tracheal papillomatosis is more problematic in children in whom it is frequently associated with laryngeal papillomatosis (18–20).

### Tracheal Stenosis

Tracheal stenosis is a dynamic pathological entity of the trachea, with tissue growing within the lumen and frequent involvements of the supporting structures. For many years prolonged intubation for mechanical ventilation and life support has been used. The widespread use of endotracheal tubes with low pressure and high volume balloons and tracheostomies in patients who need prolonged assisted ventilation has diminished, but not eliminated, the complications of respiratory support. Today, endotracheal intubation is the most frequent cause of tracheal stenosis.

Tracheal stenosis can be at one or at various levels. The same endotracheal tube used to treat tracheal stenosis can be the cause of laryngeal obstruction as a consequence of edema, granulomas or erosions (21,22). The balloon can cause ischemia and necrosis of the mucosa and cartilage. This can lead to the formation of granulation tissue and scarring that cause circumferential stenosis. This type of lesion is usually located between the fourth and seventh tracheal rings. Less frequently, an endotracheal tube that is not well centered and consequently repeatedly irritates

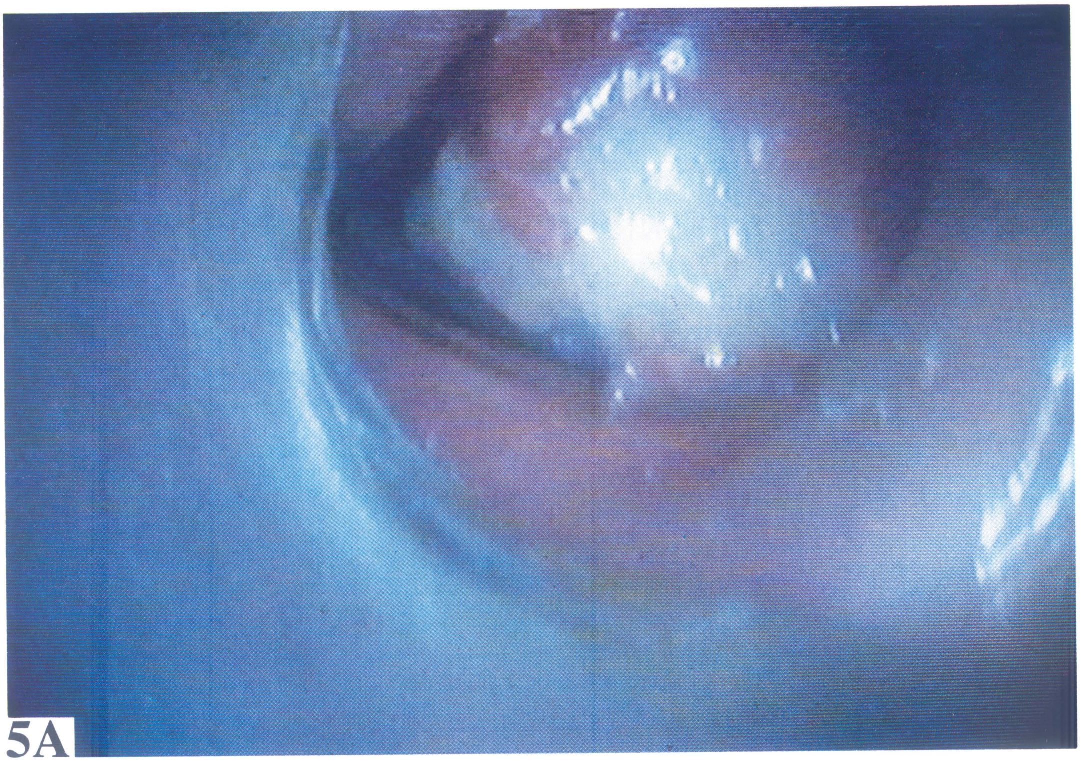

**Figure 5a** Endobronchial tumor before treatment.

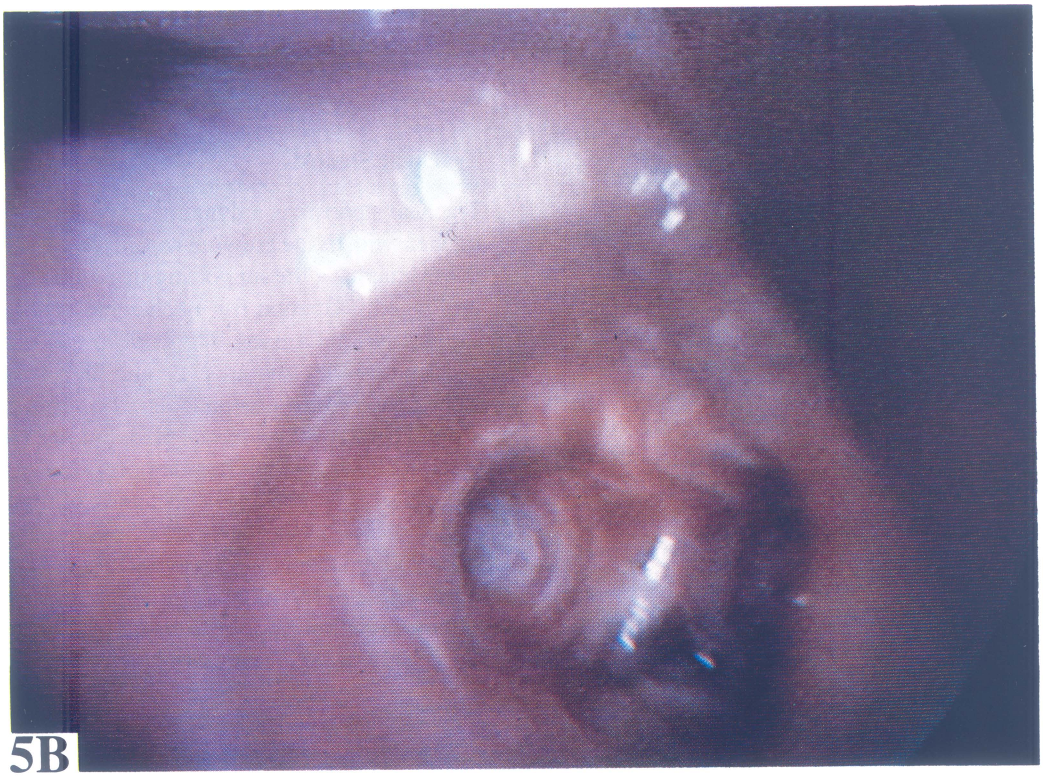

**Figure 5b** Same tumor after treatment.

and erodes the mucosa can lead to the formation of granulomas at the edge of a tracheotomy, at the bend of the cannula, or at the distal tip of the tube and this can progress to stenosis. Infection, loss of cartilaginous support and tracheomalacia exacerbate these processes.

Treatment aims to reestablish the airway by endoscopic procedures or by an external surgical approach. Surgical procedures such as resection of the affected rings and end-to-end anastomosis, tracheoplasty, etc., carry certain potential risks not usually associated with endoscopic resection. These include an increased anesthesia time, prolonged hospitalization, loss of blood and problems related to the transfusion, and postoperative discomfort of the patient. Repeated procedures, if needed, are difficult.

Endoscopic methods include traditional dilatation, which in itself is not free of risk, prolonged placement of a fixed dilating prothesis, the use of lasers, or combined laser and stenting (Fig. 6) The treatment of tracheal stenosis with a laser treatment is simple and results can be spectacular. The airway is reestablished in a few minutes, independent of the grade of obstruction or of location.

This makes laser treatment the fastest and safest method for tracheal stenosis. Unfortunatelly, many of these reopened stenoses will not remain open for more than 2 months because the mechanism for formation of tracheal stenosis involves not only the growth of granulation or fibrotic tissue into the tracheal lumen, but also a failure of the supporting structures. The conclusion of many authors (7–9,23–25) is that the best results are obtained in patients with diaphragmatic stenosis extending less than 1 cm along the longitudinal axis of the trachea and with no destruction of the wall structures. In these patients, the effects of treatment of the stenosis are usually permanent. In patients with more complex stenosis, the recurrence rate varies between 50 and 75% (23–26). The recommendation is to always try laser treatment first and, if there is recurrence, proceed to surgical correction or stent placement (27–32).

### Complications, Accidents and Their Prevention

Massive hemorrhage with resulting hypoxemia and perforation of surrounding structures are the most serious complications that can be encountered during laser photoresection. These can be avoided in the majority of cases with good patient selection and technique. The most serious risk derives from poor ventilation. In essence, many of these patients have serious ventilatory insufficiency or failure consequent to the obstruction that has motivated the treatment. Therefore, the principal objective is a rapid tracheal or bronchial reopenning that is enough to resolve the functional respiratory problem. A timid or insufficient attempt at reopening can accentuate the obstruction (8).

When significant hemorrhage is produced during treatment, steps are taken to contain the hemorrhage and to keep the airways well ventilated. If bleeding is produced by a lesion situated in the trachea, we pass the lesion with the rigid bronchoscope. Our goal is to initially compress the point of hemorrhage and ventilate the bronchial tree. We later coagulate with the laser. With bronchial lesions, we withdraw the bronchoscope to the trachea to ventilate the contralateral side while we aspirate the blood out to the airway and try to stop the hemorrhage with local intervention such as using the laser for coagulation.

Perforation of surrounding structures is very rare, but when it does occur, it is usually catastrophic. It is best to avoid this by using good technique, avoiding direct laser shots to the tracheal or bronchial walls and not stubbornly continuing the resection when the involved tissue does not absorb the laser beam well since this can be deeply damaging the surrounding structures (7,33,34).

The mortality rate during treatment varies according to the reported series. It is lower among those using a rigid bronchoscope (7–9) than in those using a fiberoptic bronchoscope (35,36). Despite the fact that some authors (15,37–39) find no difference in the results with both methods, we believe the margin of safety with the rigid bronchoscope is superior to the flexible bronchoscope. This is because of better visibility and the ability to introduce aspiration tubes and forceps, effect compression, and incise tissues with the laser. Also, the time needed for the intervention is less. The flexible bronchoscope can be introduced through the rigid bronchoscope to treat more distal areas. All these make treatment with a rigid bronchoscope superior to using a flexible bronchoscope alone. Risks are further minimized using laser energies of no greater than 45 watts and by maintaining oxygen levels of less than 50% during laser emission. There are several reports like that of Casey and colleagues (41), of an endotracheal ignition of the fiberbronchoscope when using high energy laser bursts in the presence of high concentrations of oxygen. Finally, other infrequent complications have been reported. These include pneumothorax or pneumomediastinum which usually occur in relation to mechanical ventilation during anesthesia, especially when jet ventilation is used (42,43). There has even been a case reported of the loss of the metallic protector of the distal end of the laser fiber because of excessive heating (44).

### CONCLUSION

Overall, laser bronchoscopy has made a significant contribution to the treatment of intraluminal airway disease. It can be used in conjunction with other forms of therapy.

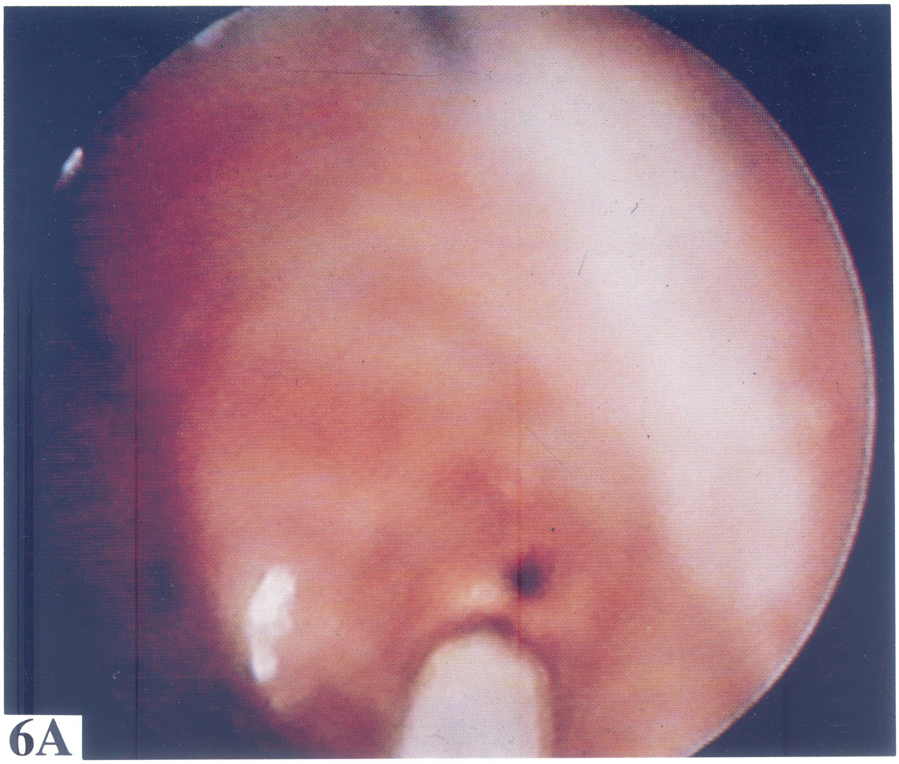

**Figure 6a** Bronchial stenosis before treatment.

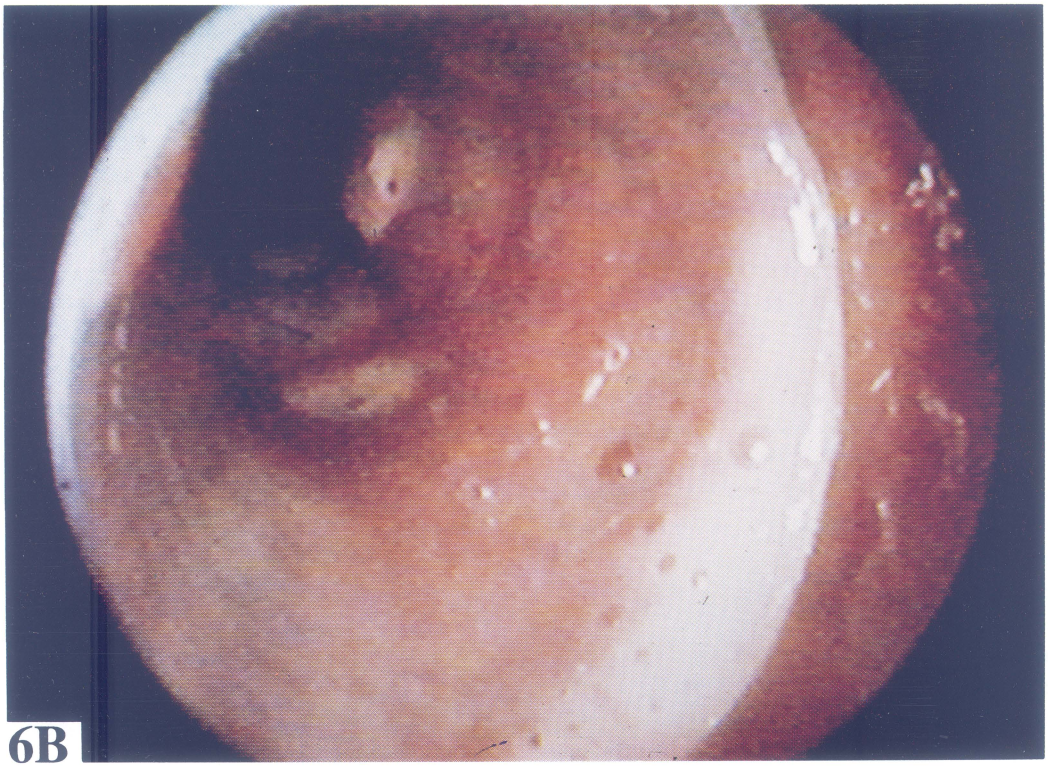

**Figure 6b** Same stenosis after photoresection and stent placement.

It can provide significant palliation in patients with malignant or benign airway tumors who are not candidates for surgery. It also may spare some patients with tracheal stenosis the need for more invasive surgery. With care taken for good patient selection and technique, laser photoresection represents a low risk option to achieve significant palliation in patients with benign and malignant conditions compromising the airway.

### REFERENCES

1. Kramer R, Som ML. Further study of adenoma of the bronchus. Ann Otol Rhinol Laringol 1935;44:861–878.
2. Som ML. Adenoma of the bronchus. Endoscopic treatment in selected cases. J Thorac Surg 1949;18:462–478.
3. Olsen AM. Carcinoma of the trachea Arch Otolaryngol 1936;30:615–630.
4. Carpenter RJ, Noel BH, Sanderson DR. Comparison of endoscopic cryosurgery and electrocoagulation of bronchi. Tr Am Acad Ophthmol Otol. 1977;84:313–323.
5. Price JC, Percapio B, Murphy PW, Henderson RL. Recurrent adenoid cystic carcinoma of the trachea. Intraluminar radiotherapy Otolaryngol Head Neck Surg 1979;87:614–623.
6. Strong MS, Vaughan CW, Polany, Wallace R. Bronchoscopic carbon dioxyde laser surgery. Ann Otol Rhinol Laryngol 1974;83:769–776.
7. Dumon JF, Shapshay S, Bourcereau J, Cavaliere S, Meric B, Garbi N, Beamis J. Principles for safety applications of Neodymium-Yag laser in bronchology. Chest 1984;86:163–168.
8. Personne C, Colchen A, Leroy M, Vourc hG, Toty L. Indications and technique for endoscopic laser resections in bronchology. J Thorac Cardiovasc Surg 1986;91:710–715.
9. Cavaliere S, Foccoli P, Farina PL. Nd:Yag Laser bronchoscopy. A five-year experience with 1396 applications in 1000 patients. Chest 1988;94:15–21.
10. Diaz-Jimenez JP, Canela Cardona M, Maestre Alcacer J, Balust Vidal M, Fontanals Tortra J, Balust Vidal J. Treatment of obstructive tracheobronchial disease with the Yag-Nd laser: 400 procedures in a 4-year experience. Med Clin 1989;93:244–248.
11. Brutinel WM, Cortese DA, McDougall JC. Bronchoscopic phototherapy with the neodymium yag laser. Chest 1984;86: 158–159.
12. Unger M. Lasers and their role in pulmonary medicine:present and future. In Fishman AP, ed Update: pulmonary diseases and disorders, New York: McGraw-Hill, 1992;419–432.
13. Emslander HP, Munteanau J, Prauer HJ, et al. Palliative endobronchial tumor reduction by laser therapy. Respiration 1987;51:73–79.
14. Dumon JF, Reboud E, Garbe N, Aucomte F, Meric B. Treatment of tracheobronchial lesions by laser photoresection. Chest 1982;81:278–284.
15. Unger M. Neodymium:yag laser for malignant and benign endobronchial obstruction. Clin Chest Med 1985;6:277–290.
16. Diaz-Jimenez JP, Canela Cardona M, Maestre Alcacer J. Nd:Yag Laser photoresection of Low Grade Malignant Tumors of Tracheobronchial Tree. Chest 1990;97:920–922.
17. Desai SJ, Metha AC, Vanderbur Medendorp S, Golish JA, Ahmmad M. Survival experience following ND:Yag laser photoresection for primary bronchogenic carcinoma. Chest 1988;94:939–944.
18. Strong MS, Vaughan CW, Healy GB, Cooperband SR, Clemente MACP. Recurrent respiratory papillomatosis. Management with the carbon dioxide laser. Ann Otol Rhinol Laringol 1976; 85:508–516.
19. Fearon B, Macrae D. Laryngeal papillomatosis in children. J Otolaryngol 1976;5:493–496.
20. Robbins KT, Woodson GE. Current concepts in the management of laryngeal papillomatosis. Head Neck Surg 1984;6:861–866.
21. Strong MS, Jako GJ. Laser surgery in the larynx. Ann Otolaryngol 1972;81:791–798.
22. Healy CG, McGill T, Strong MS. Surgical advances in the treatment of lesions in the pediatric airway. The role of the carbon dioxide laser. Pediatrics 1978;61:380–383.
23. Personne CL, Colchen A, Toty L. Le laser Yag-Nd en bronchologie. Sa place dans la pathologie non cancereuse. Ann Oto-Laryng 1985;102:65–68.
24. Dumon JF, Reboud E, Guidicelli R, Fuentes P, Meric B. Interet du laser dans les stenosis tracheales non tumoraux. A propos de treinte malades. Ann Chir 1981;35:620–622.
25. Ossof RH, Tucker GF, Duncavage JA, Toohill RJ. Efficacy of bronchoscopic carbon dioxide laser surgery for bening strictures of the trachea. Laryngoscope 1985;95:1220–1223.
26. Personne C, Colchen A, Toty L, Beretti E. Indications et limites de la resection endoscopique par laser dans les stenoses ss de la tracheales (et leur recidive apres la chirurgie). Ann Chir Thorac 1981;35:619–620.
27. Diaz-Jimenez JP, Farrero Mu~oz E, Martinez Ballarin JI, Kovitz K. Silicone stents in the management of obstructive tracheobronchial lesions: 2 year experience. J Bronch 1994;1:15–18.
28. Dumon JF. A dedicated tracheobronchial stent. Chest 1990;97:328–332.
29. Freitag L, Firusian N, Stamitis G, Greshchuchna D. The role of bronchoscopy in pulmonary complications due to mustard gas inhalation. Chest 1991;100:1436–1441.
30. Prakash UBS. Chemical warfare and bronchoscopy. Chest 1991;100:1486–1487.
31. Nashef SAM, Dromer C, Valley JE, et al. Expanding wire stents in benign tracheobronchial disease: Indications and complications. Ann Thorac Surg 1992;54:937–940.
32. Metha AC, Lee FYN, Cordasco EM, Kirby T, et al. Concentric tracheal and subglotic stenosis. Management using the Nd:Yag laser for mucosal sparing by gentile dilatation. Chest 1988;94:939–944.
33. Hetzel MR, Smith SGT. Endoscopic palliation of tracheobronchial malignancies. Thorax 1991;46:325–333.
34. Diaz-Jimenez JP, Dumon JF. Endoscopia Respiratoria y Laser. Barcelona: Ars Llibris 1991.
35. Brutinel WM, Cortese DA, Edell ES, McDougall JC, Prakash UBS, Complications of Nd:Yag laser therapy. Editorial. Chest 1988;94:902–903.
36. Beamis JF, Rabeiz EE, Vargos K, Shapshay SM. Endoscopy laser therapy for obstructing tracheobronchial lesions. Ann Otol Rhinol Laryngol 1991;100:413–419.
37. McDougall JC, Cortese DA. Neodymium Yag-Laser therapy of malignant airways obstructions: a preliminary report. Mayo Clin Proc 1983;58:35–39.
38. Wolfe WG, Sabiston DC. Management of benign and malignant lesions of the trachea and bronchi with the neodymium-ytrium-alluminium-garnet laser. J. Thorac Cardiovasc Surg 1986;91:40–45.
39. Brutinel WM, Cortese DA, McDougall JC, Gillio RG, Sergstralh EJ. A two year experience with neodymium yag laser in endobronchial obstruction. Chest 1987;91:159–165.
40. Kwale PA, Eichenhorn MS, Radke JR, Miks V. Yag laser photoresection of lesions obstructing the central airways. Chest 1985;87:283–288.
41. Casey KR, Fairfaix WR, Smith SJ, Dixon JA. Intratracheal fire ignited by the yag-Nd laser during treatment of tracheal stenosis. Chest 1983;84:295–296.
42. Vourc'h G, Fischler M, Michon F, Melchior JC, Seigneuer F. High frequency jet ventilation versus manual jet ventilation during bronchoscopy in patients with tracheobronchial stenosis. Br J Anaesth 1983;55:969–972.
43. Warner ME, Warner MA, Leonard PF. Anaesthesia of Nd-Yag laser resection of ma;or airway obstructing tumors. Anaesthesiology 1984;60:232–235.
44. Metha AC, Golish JA, Livingston DR. Loss of fiberoptic laser tip. Chest 1985;88:798.

